# The omega-3 fatty acid, eicosapentaenoic acid (EPA), prevents the damaging effects of tumour necrosis factor (TNF)-alpha during murine skeletal muscle cell differentiation

**DOI:** 10.1186/1476-511X-7-24

**Published:** 2008-07-18

**Authors:** Peter Magee, Stephen Pearson, Jeremy Allen

**Affiliations:** 1 Biomedical Sciences Research Institute, University of Salford, Manchester, M5 4WT, UK; 2 Centre for Rehabilitation and Human Performance Research, University of Salford, Manchester, M5 4WT, UK

## Abstract

**Background:**

Eicosapentaenoic acid (EPA) is a ώ-3 polyunsaturated fatty acid with anti-inflammatory and anti-cachetic properties that may have potential benefits with regards to skeletal muscle atrophy conditions where inflammation is present. It is also reported that pathologic levels of the pro-inflammatory cytokine tumour necrosis factor (TNF)-α are associated with muscle wasting, exerted through inhibition of myogenic differentiation and enhanced apoptosis. These findings led us to hypothesize that EPA may have a protective effect against skeletal muscle damage induced by the actions of TNF-α.

**Results:**

The deleterious effects of TNF-α on C2C12 myogenesis were completely inhibited by co-treatment with EPA. Thus, EPA prevented the TNF-mediated loss of MyHC expression and significantly increased myogenic fusion (*p *< 0.05) and myotube diameter (*p *< 0.05) indices back to control levels. EPA protective activity was associated with blocking cell death pathways as EPA completely attenuated TNF-mediated increases in caspase-8 activity (*p *< 0.05) and cellular necrosis (*p *< 0.05) back to their respective control levels. EPA alone significantly reduced spontaneous apoptosis and necrosis of differentiating myotubes (*p *< 0.001 and *p *< 0.05, respectively). A 2 hour pre-treatment with EPA, prior to treatment with TNF alone, gave similar results.

**Conclusion:**

In conclusion, EPA has a protective action against the damaging effects of TNF-α on C2C12 myogenesis. These findings support further investigations of EPA as a potential therapeutic agent during skeletal muscle regeneration following injury.

## Background

Skeletal muscle can be affected by a number of potentially damaging conditions which lead to muscle atrophy. These conditions can include ageing and disease states. With ageing, muscle wasting occurs which is a chronic condition complicated by multitude of factors. A number of models have been suggested to explain the ageing related loss of muscle tissue, amongst which is the immunological theory. Here changes in DNA methylation and mutation of the somatic cells (epigenetic, chromosome abnormalities) could lead to increases in autocatalytic processes which ultimately leads to self destruction. Hence, a gradual cumulative effect here, such as an immune inflammatory response may lead to muscle damage and sarcopenia [1].

Disease states can be both chronic and acute, but both ageing and disease can share some common factors. In particular, inflammation has been suggested to be present in a number of disease states associated with muscle atrophy, such as cancer, heart failure, rheumatoid arthritis, chronic obstructive pulmonary disease, HIV/AIDS, and also ageing related muscle wasting. Associated with this response are pro-inflammatory cytokines, such as tumour necrosis factor (TNF)-α that are commonly present at elevated levels (0.5–10 ng/ml) during disease[2-5]. A bimodal response to TNF-α has been reported. Whereas, pathologic levels of TNF-α have been identified as playing a significant role in the mechanisms associated with skeletal muscle wasting [6], low physiological concentrations (0.05 ng/ml) appear to activate myogenesis [7]. A number of previous studies, both *in vitro *and *in vivo *have shown that raised levels of TNF-α causes increased muscle loss [8-12]. At least two mechanisms may account for the skeletal muscle-wasting effects of TNF-α: inhibition of myogenesis in myoblasts; apoptosis of myoblasts and myotubes. A number of *in vitro *studies suggest the effects of TNF-α are specific to the stage of myotube differentiation at the time of administration. Thus, delivery of TNF-α to primary human myoblasts or murine C2C12 myoblasts inhibits myosin heavy chain (MyHC) expression and myogenic differentiation [13-16] whereas treatment of differentiated myotubes with TNF-α appears to have marginal effects on their total or MyHC protein content [13,16]. However, a more recent finding suggests differentiated (C2C12) myotubes are susceptible to TNF-α-mediated apoptosis [17].

The cellular action of TNF-α is complex and is exerted through a number of signalling pathways in skeletal muscle. Recent findings suggest that low levels of TNF-α are required for myoblast proliferation and this aspect of myogenesis is regulated by activation of p38 mitogen-activated protein kinase (MAPK) [7]. In contrast, at pathologic levels of TNF-α, high levels of myoblast apoptosis are observed and myogenic differentiation is inhibited [18,19]. The apoptosis observed during differentiation of myoblasts is characterized by initial NF-κB activation [16,19] and activation of caspase-8 [19]. In differentiated myotubes, pathologic levels of TNF-α have been found to increase total and myofibrillar protein content through stimulation of MAPK pathways [20], however these anabolic effects are counteracted by findings that, at high levels, TNF-α also induces myotube apoptosis which is characterized by enhanced caspase-3 activity [17].

A number of experimental and clinical studies have described potential health benefits for ώ-3 polyunsaturated fatty acids (PUFA), notably in reducing the incidence of cardiovascular disease [21]. Eicosapentaenoic acid (EPA) is a ώ-3 PUFA with demonstrable anti-inflammatory activities that may have potential benefits with regards to atrophic skeletal muscle conditions [22]. In this regard, it has been reported that in a murine model of cachexia, EPA treatment caused a reduction in the rate at which skeletal muscle protein was lost [23] and that preservation of skeletal muscle protein was due to downregulation of the ubiquitin-proteasome proteolytic pathway [24]. EPA treatment has also been shown to attenuate the proteolytic and apoptotic effects of a cachectic factor in fully differentiated myotubes from the murine C2C12 myogenesis model, although TNF-α was not used in these studies [25,26].

These findings led us to hypothesize that EPA may have a protective effect against skeletal muscle damage induced by pro-inflammatory TNF-α. Hence, the aims of the present study were to utilise TNF-α in a damage model of murine C2C12 myogenic differentiation and to determine whether EPA treatment was able to reduce the deleterious effects of TNF-α on skeletal muscle cell differentiation. We evaluated the responses of differentiating cells to TNF-α and EPA treatments by morphological criteria and by expression of MyHC and quantified their effects on apoptosis by measuring caspase-8 activity.

## Results

### Effect of TNF-α and EPA on Myogenic Differentiation

**Figure 1:**
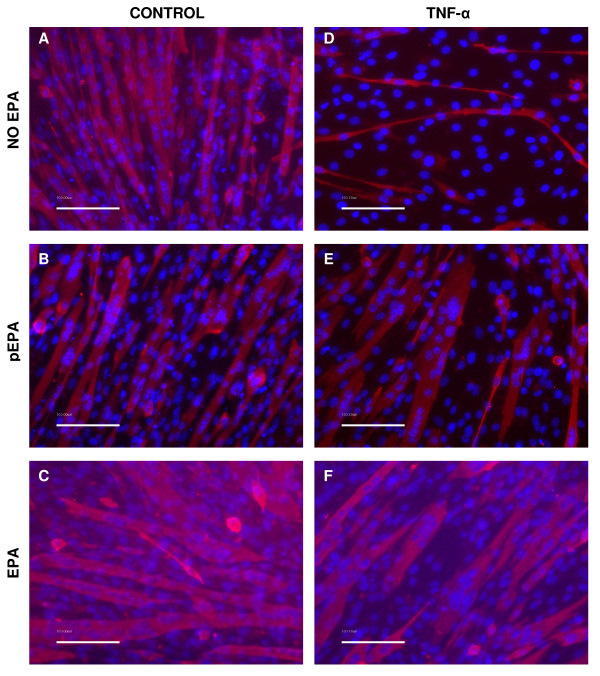
**EPA ameliorates the effects of TNF-α on differentiating myotube appearance and MyHC expression**. C2C12 cells were induced to differentiate in DM in the presence or absence of TNF-α (20 ng/ml). EPA (50 μM) was added together with TNF-α as a co-treatment (EPA). Alternatively, for some experiments EPA was administered alone for a 2 hour pre-treatment (pEPA) after which it was withdrawn and replaced by TNF-α alone in DM. Incubations were continued for 5 days in DM with replenishment of EPA and TNF-α at media changes. Representative images show immunofluorescence detection of Alexa-Fluor546 conjugated anti-MF20 antibody against MyHC (pink) and DAPI counterstained nuclei (blue) for various treatments with EPA and TNF-α. Control treatments are shown in the left panel images and TNF-α treatments in the right panel images. Calibration bars are 100 μm.

Under permissive conditions in a low-serum culture medium (DM), C2C12 myoblasts will differentiate to form myotubes. After 5 days following initiation of differentiation by incubation in DM, the morphological appearance of formed myotubes was examined (figure 1). Under control growth conditions in DM, fully formed myotubes were prominent throughout (figure 1A). EPA alone, either administered as a 2 h pre-treatment in DM (figure 1B), or continuously throughout the duration of the experiment (figure 1C), had no apparent effect on the normal pattern of myogenesis; myotubes were formed, their rate of differentiation was similar to controls (data not shown) and they appeared morphologically indistinct from controls (figure 1A); the timing of the appearance of MyHC (data not shown) and its pattern of expression as visualised by immunocytochemistry, was also similar to that of controls (figure 1A). Previous studies have examined the effects of the inflammatory cytokine TNF-α on myotube formation and established that high concentrations, typically found in chronic disease states, are able to reversibly inhibit myotube formation. We first confirmed that TNF-α (20 ng/ml) markedly inhibited the formation of myotubes (figure 1D). We then examined the effect of 50 μM EPA co-treatment together with TNF-α on myotube differentiation. Data shown in figure 1F indicate that EPA was able to prevent the deleterious effects of TNF-α on myotube differentiation and the pattern of MyHC expression. Furthermore, a single dose of EPA given for 2 h, prior to treatment with TNF-α alone, was sufficient to be able to largely prevent these inhibitory actions of TNF-α on myogenesis (figure 1E).

**Figure 2:**
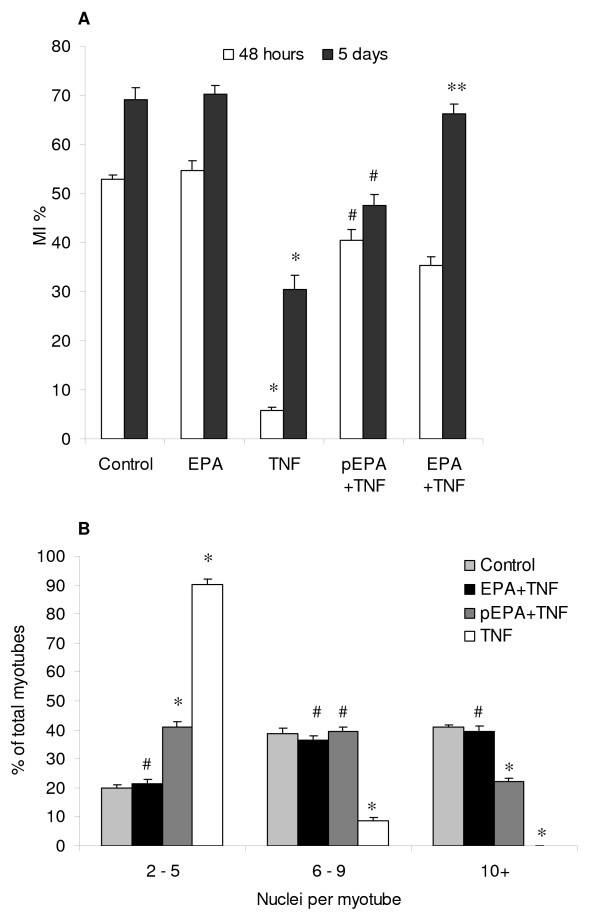
**EPA reverses TNF-α-mediated interference with myoblast fusion**. C2C12 cells were induced to differentiate in the presence or absence of TNF-α (20 ng/ml) and EPA (50 μM). EPA was added together with TNF-α as a co-treatment (EPA+TNF) or, alternatively EPA was administered alone for a 2 hour pre-treatment after which it was withdrawn and replaced by TNF-α alone in DM (pEPA+TNF). Incubations were continued for 48 hours or 5 days in DM with replenishment of EPA and TNF-α at media changes. A myogenic index (MI) of fusion was calculated from ten images of randomly chosen microscope fields for DAPI and MyHC stained cells from each treatment. The total number of nuclei and the number of nuclei incorporated into myotubes were counted (A). Myotube size heterogeneity was evaluated after 5 days from the same images by calculating the number of nuclei per myotube and classifying them arbitrarily to categories of small (2–5 nuclei), medium (6–9 nuclei) or large (>10 nuclei) myotubes (B). Data are expressed as means ± standard error of mean (SEM) from 3 independent experiments (*p < 0.05 v. respective control; ^#^*p *< 0.05 v. TNF-α; **NS v. control).

**Figure 3:**
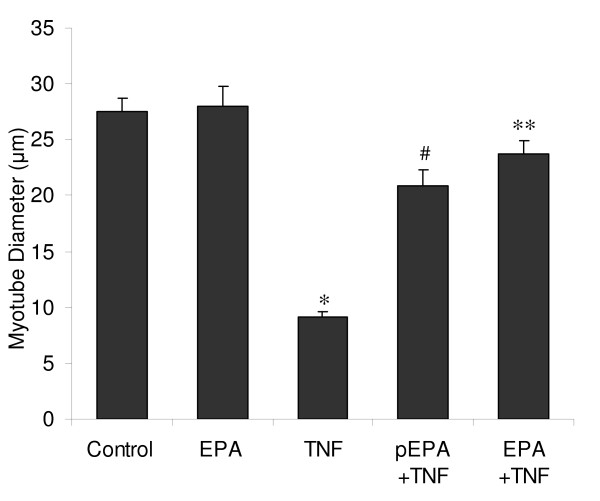
**EPA prevents a TNF-α-mediated reduction in myotube size**. C2C12 cells were induced to differentiate in the presence or absence of TNF-α (20 ng/ml) and EPA (50 μM). EPA was added together with TNF-α as a co-treatment (EPA+TNF) or, alternatively EPA was administered alone for a 2 hour pre-treatment after which it was withdrawn and replaced by TNF-α alone in DM (pEPA+TNF). Incubations were continued for 5 days in DM with replenishment of EPA and TNF-α at media changes. Myotube diameters were calculated for DAPI and MyHC stained cells from each treatment. Data are expressed as means ± standard error of mean (SEM) from 3 independent experiments (*p < 0.05 v. control; ^#^*p *< 0.05 v. TNF-α; **NS v. control).

These protective effects of EPA were further evidenced when we investigated morphological parameters of myogenesis; namely myoblast fusion (Figure 2) and myotube size (Figure 3). The results of a myoblast fusion index (MI) presented in figure 2A show that TNF-α significantly inhibited (*p *< 0.05) MI after 48 hours and at 5 days, compared to control treatment in DM. EPA alone had no significant effect on the MI. EPA also had no effect on the rate of differentiation as indicated by a comparison between EPA and control after 48 hours of treatment. Treatment with EPA, either as a pre-treatment or as a co-treatment with TNF-α, improved the MI with additional benefit derived from a longer incubation with EPA. Following pre-incubation with EPA, the MI was partially restored with levels significantly higher than with TNF-α, reaching 40% (*p *< 0.05) and 50% (*p *< 0.05), compared to 5% and 30% for TNF-α, after 48 hours and 5 days respectively. When EPA was added as a co-treatment with TNF-α, the MI was partially restored after 48 hours but was fully restored to control levels of 70% by 5 days (NS v. control). In figure 2B, data are shown for the average number of nuclei found per myotube after 5 days, as a marker of the heterogeneity of myotube size. Myotubes treated with TNF-α showed the greatest uniformity but were much smaller and poorly developed, with >90% having <6 nuclei, significantly higher than controls at 20% (*p *< 0.001). EPA co-treatment completely prevented these effects of TNF-α and the size distribution of myotubes was not significantly different from that of controls. Here, most myotubes were large with 40% having >10 nuclei, compared to 0% with TNF-α treatment (*p *< 0.001). EPA pre-treatment was able to partially reverse the effects of TNF-α and here myotubes were more heterogeneous, with proportionately more smaller myotubes and fewer large myotubes compared to controls (*p *< 0.001). The results presented in figure 3 show that TNF-α significantly inhibits myotube size (*p *< 0.05), reducing mean diameter after 5 days from approximately 27 μm to 8 μm, compared to control. EPA alone did not significantly alter myotube size. However, treatment of differentiating myotubes with EPA, either as a pre-treatment or as a co-treatment, with TNF-α improved myotube size. Following pre-incubation with EPA, myotube size was significantly higher than with TNF-α (*p *< 0.05), reaching approximately 20 μm. When EPA was added as a co-treatment with TNF-α, myotube size was also significantly increased compared to TNF-α (p < 0.05) reaching approximately 24 μm, a size not significantly different from controls (NS v. control).

### Effects of EPA on cellular necrosis

**Figure 4:**
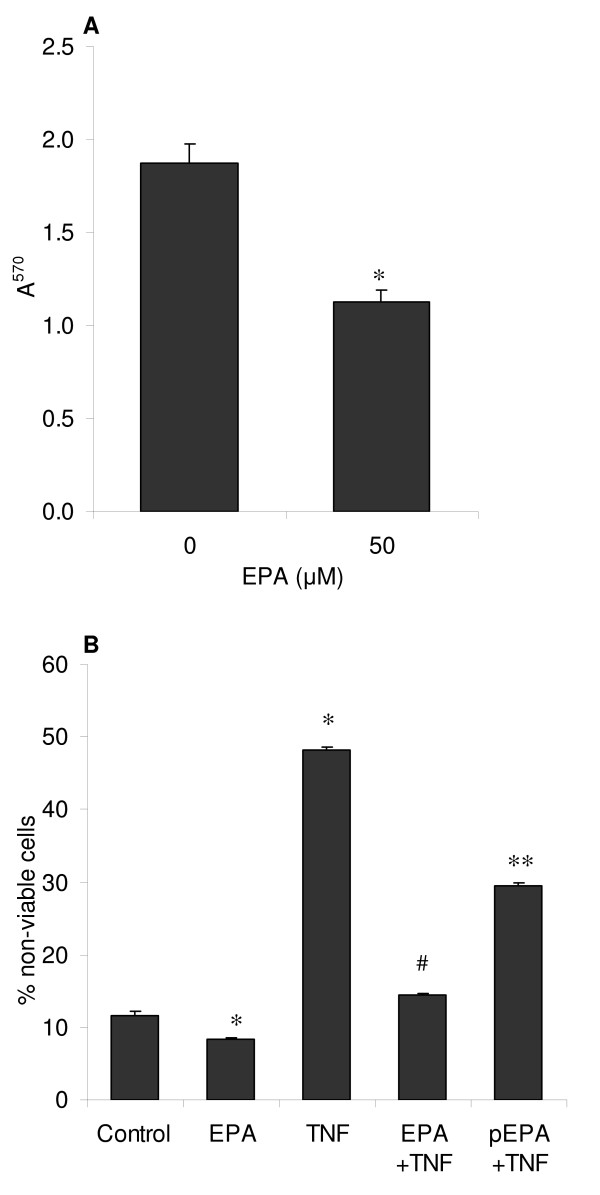
**EPA ameliorates baseline and TNF-α-induced cellular necrosis**. Any potential cytotoxic effect of EPA on C2C12 myotube formation was investigated after 5 days following induction of differentiation by DM using a CellTiter Blue assay to detect all non-viable cells (A). The effect of TNF-α (20 ng/ml) on cellular necrosis and the ability of EPA to block this activity were also assessed by Trypan Blue dye exclusion to detect necrotic cells (B). Here, EPA was added together with TNF-α as a co-treatment (EPA+TNF) or, alternatively EPA was administered alone for a 2 hour pre-treatment after which it was withdrawn and replaced by TNF-α alone in DM (pEPA+TNF). Incubations were continued for 5 days in DM with replenishment of EPA and TNF-α at media changes. Data are expressed as means ± standard error of mean (SEM) from 3 independent experiments (*p < 0.05 v. control; ^#^NS v. control; **p < 0.05 v. TNF-α).

Since it has been reported that non-esterified free fatty acids can have cytotoxic effects on cultured cells [27], we used EPA conjugated with BSA in all studies. To investigate the effects of EPA on cell death in differentiating C2C12 myotubes, several complementary assays were performed. A commercial CellTiter-Blue cytotoxicity assay was performed to determine cell death over 5 days of culture in DM. This assay does not distinguish between the mode of cell death i.e. apoptosis or necrosis. As shown in figure 4A, 50 μM EPA significantly reduced (*p *< 0.05) cell death in DM, compared to controls. These data suggested EPA was protective against cell death during myotube formation. Using an alternative approach of dye exclusion (Trypan Blue) which will not detect cells in the earlier stages of apoptosis with intact cell membranes, we confirmed that EPA significantly reduced cellular necrosis (*p *< 0.05) at an earlier time-point (48 h) from initiation of differentiation (figure 4B). We next examined whether TNF-α induced cellular necrosis in this model and if EPA treatment could prevent this activity (figure 4B). Preliminary experiments suggested that significant TNF-α-mediated cell necrosis became apparent by 48 hours (data not shown), so this time-point was adopted for the remainder of the experiment. TNF-α (20 ng/ml) induced significant (*p *< 0.05) cellular necrosis by 48 hours after initiation of differentiation, compared to control with almost 50% of cells assessed as non-viable. EPA treatment was found to have a protective effect against the TNF-α-induced increase in necrosis. Co-treatment with EPA completely abolished (p < 0.05) the effects of TNF-α, compared to control (Fig 4B), whereas a 2 hour pre-incubation of cells with EPA, prior to treatment with TNF-α alone, was sufficient to partially but significantly reduce (*p *< 0.05) the TNF-α-induced damage, compared to TNF-α alone (Fig 4B).

### Effect of EPA on apoptosis

**Figure 5:**
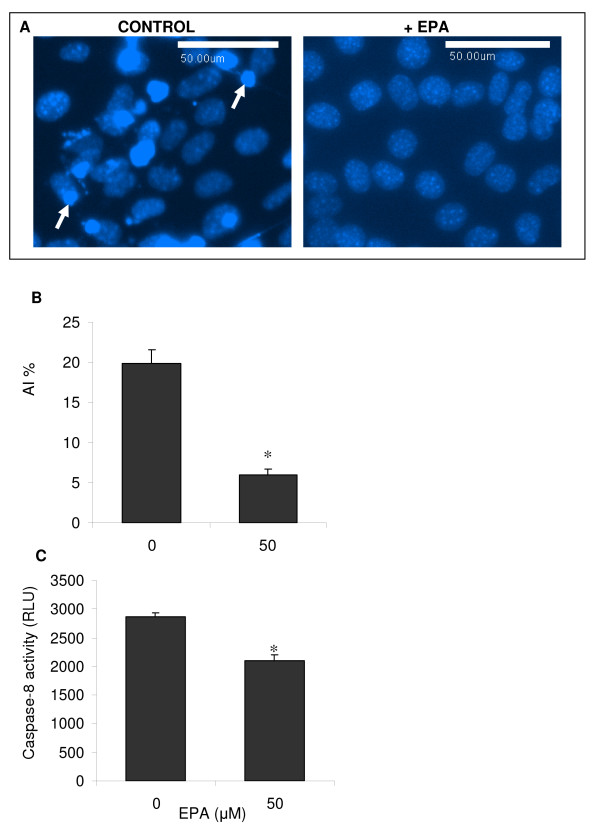
**EPA blocks baseline C2C12 apoptosis**. C2C12 cells in DM for 5 days were stained with Hoechst 33342 to identify brightly fluorescent, condensed apoptotic nuclei. Representative images are shown in (A) for cells in the presence or absence of 50 μM EPA with arrows indicating example apoptotic nuclei. A semi-quantitative analysis was performed by calculating an apoptosis index (AI%) from these images (B). Quantitative analysis of caspase-8 activity (measured in relative light units, RLU) from cultures in the presence or absence of EPA was evaluated using the Caspase Glo-8 commercial assay (C). Data are expressed as means ± standard error of mean (SEM) from 3 independent experiments. (*p < 0.05 v. control). Calibration bars are 50 μm.

**Figure 6:**
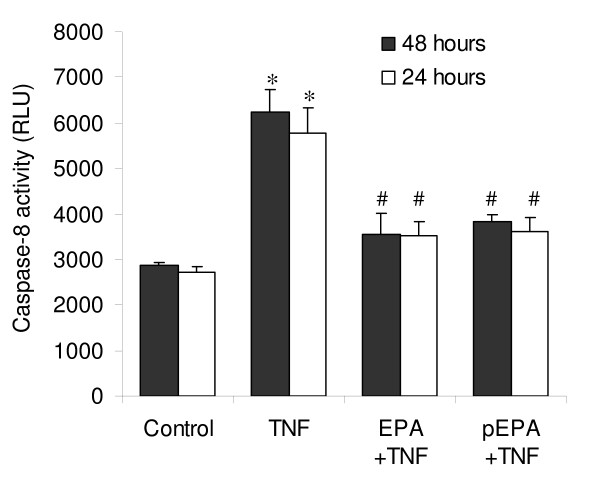
**EPA inhibits the TNF-α-induced caspase-8 activity associated with apoptosis**. The nature of the pro-apoptotic effect of TNF-α on C2C12 myotube formation and its prevention by EPA were examined after 24 or 48 hours in DM by evaluating caspase-8 activation (measured in relative light units, RLU) using the Caspase Glo-8 commercial assay. EPA was added together with TNF-α as a co-treatment (EPA+TNF) or, alternatively EPA was administered alone for a 2 hour pre-treatment after which it was withdrawn and replaced by TNF-α alone in DM (pEPA+TNF). Data are expressed as means ± standard error of mean (SEM) from 3 independent experiments. (*p < 0.05 v. respective control; ^#^NS v. respective control).

Further investigation of the apparent protective effect of EPA against the inhibition of myogenesis in differentiating C2C12 myotubes by TNF-α was carried out to determine whether protection against apoptosis was conferred by EPA. We first examined whether EPA had an effect on baseline levels of apoptosis. When 50 μM EPA was administered to C2C12 cells in DM there was apparent suppression of apoptosis measured after 5 days indicated by Hoechst 33258 staining (figure 5A). A calculated apoptosis index (AI) showed a significant >50% reduction (*p *< 0.001) in the proportion of apoptotic cells from approximately 20% to 6% following treatment with EPA, compared to untreated controls (figure 5B). To confirm these findings and to try and determine the nature of the protective effect we measured caspase-8 activity using a commercial assay (Caspase Glo-8, Promega) because in a similar model system, transfer of C2 myoblasts to DM has previously been reported to provoke spontaneous apoptosis mediated by caspase-8 activity [19]. Treatment of cells in DM with 50 μM EPA significantly inhibited (*p *< 0.05) the caspase-8 activity associated with spontaneous apoptosis, compared to untreated controls (figure 5C). We next investigated whether EPA could inhibit TNF-α-mediated apoptosis. Since TNF-α-induced apoptosis is associated with caspase-8 activation, by mechanisms downstream of the TNF-α receptor 1 (TNFR1) in differentiating myoblasts [18,19,28], we evaluated caspase-8 activity in response to treatment with TNF-α, and with EPA to determine whether EPA could block the caspase-8 activity associated with TNF-α-induced apoptosis (figure 6). In response to TNF-α (20 ng/ml) there was a significant (*p *< 0.001) increase in caspase-8 activity by 24 or 48 hours in DM, compared to respective untreated controls. EPA treatment was found to block the TNF-α-induced increase in caspase-8 activation at both of these time-points. Co-treatment with EPA completely blocked (*p *< 0.05) the effects of TNF-α on caspase-8 activity. A similar significant (*p *< 0.05) protective effect of EPA was obtained when cells were instead pre-incubated for 2 hours with EPA, prior to treatment with TNF-α alone.

## Discussion

It has previously been suggested that muscle cells exposed to TNF-∝ during differentiation will be adversely affected. This study examined the potential for EPA to protect skeletal muscle cells during their differentiation from myoblasts to myotubes in an inflammatory damage model. The main findings of this study were that EPA has a significant and protective effect on myogenesis in a murine C2C12 model of muscle damage. Undifferentiated C2C12 myoblasts, the in vitro equivalent of satellite cells, can be induced to differentiate into non-proliferating, multinucleated, fully differentiated myotubes over a period of days in the presence of a reduced-serum growth medium. Using this model, or primary human myoblasts, it has been shown that TNF-α has differential effects according to the developmental stage of myogenesis and these are mediated through alternative mechanisms that inhibit myotube formation or that induce myoblast and myotube apoptosis. Thus, in cultured differentiating primary human or C2C12 myoblasts, addition of TNF-α completely inhibits MyHC expression and myogenic differentiation [13,14,29,30]. In this study, using the C2C12 model, we observed reductions in indices of myogenesis in response to TNF-α treatment, including myotube size, myoblast fusion index and expression of MyHC. However, the presence of EPA blocked the TNF-induced inhibition of MyHC expression. EPA treatment by itself does not seem, for the limited range of markers we have measured, to significantly affect the differentiation process except in relation to baseline cell death. It is possible to speculate that this could potentially enhance differentiation by increasing the pool of viable myonuclei available that can contribute to myotube formation. Our results showed a significant (*p *< 0.05) effect of EPA on the reduction of myogenesis caused by TNF-α, that was evident in both the continuous and pre-incubation conditions. Alone, TNF-α caused a 9-fold decrease in MI after 48 hours whereas by 5 days this was only 2-fold. These data are consistent with reports that more mature myotubes are less susceptible to the damaging effects of TNF-α on myogenesis [13,14]. In conjunction with this observation, the fusion index was markedly improved in the presence of EPA, such that differentiating myotubes continuously dosed with both TNF-α and EPA were significantly different to those dosed with TNF-α alone with MI showing no alteration from control levels of 70% by day 5. Similar effects were noted on indices of myotube size whereby myotube diameter and heterogeneity of size closely matched those of controls. EPA delivered as a short pre-treatment was still effective against TNF-α but these parameters were only partially restored to their respective control levels. This observed increased efficacy of EPA co-treatment most likely reflects the continuous availability of EPA during the experiments and was also a feature in the viability experiments and in apoptosis experiments and is discussed further below.

Following TNF-α treatment, there is an initial 24 h period of myoblast proliferation associated with transient NF-κB and Jun kinases (JNK) 1 and 2 activation, followed by induction of apoptosis [19]. More recent findings show direct apoptotic activity of TNF-α on formed myotubes mediated by caspase-3 activation [17]. Here, our data have shown that caspase-8 activation is associated with the prevention of normal C2C12 myotube differentiation by TNF-α. Although this is consistent with previous findings showing that caspase-8 activation was an essential requirement for TNF-α induced apoptosis in this model [19], a mechanism most likely mediated through the TNF-receptor 1 (TNF-R1) signalling complex, this requires further confirmation. In the background it was stated that TNF-α can inhibit differentiation in at least two ways; inhibition of myogenesis in differentiating myoblasts; induction of apoptosis of myoblasts and myotubes. Our data support a contribution to enhanced differentiation by decreasing TNF-mediated signalling through pathways involving the activation of caspase-8. Our data showed that the effects of TNF-α on caspase-8 were ablated following both a 2 hour pre incubation and continuous administration of 50 μM EPA, suggesting EPA as a potent inhibitor of TNF-α activated caspase-8 activity. In support of our findings, in fully differentiated C2C12 myotubes PIF-mediated caspase activation and apoptosis can be attenuated by EPA pre-treatment [26]. Although TNF-α was not used in the reported study, those findings lend support to a view that the protective actions of EPA may be independent of differentiation status, although further studies will be required to confirm this. There are several previous reports that docosahexaenoic acid (DHA) the other primary ώ-3 PUFA found in fish oils, attenuates human monocyte apoptosis or murine fibrosarcoma necrosis induced by TNF-α [27,31]. In general agreement with these studies it was observed here that EPA treatment had a beneficial effect against the TNF-α-induced increase in necrosis. Whereby, EPA completely eliminated the effects of TNF-α on cell death, and a 2 hour pre-treatment of cells with EPA, prior to TNF-α dosing, was sufficient to partially, but significantly reduce (*p *< 0.05) the TNF-α-induced damage, compared to TNF-α alone. However, as discussed above, the pre-treatment was sufficient to block caspase-8 activity induced by TNF-α. This may reflect an increased susceptibility of pathways associated specifically with caspase activation, to EPA inhibition, although this will require further clarification. A 2 hour pre-treatment with EPA has been used previously and found to effectively inhibit apoptosis in fully differentiated myotubes [26]. Other reported in vitro EPA dosing regimens vary from 30 minutes [32] to 24 hours pre-treatment [27,33,34]. 24 hours EPA treatment was sufficient to significantly alter the membrane lipid composition of macrophages; EPA increased from 0.3 to 22.7% and AA fell from 7.3 to 2.0% [35]. It is possible to speculate that increasing the pre-treatment time from 2 hours up to 24 hours would increase the efficacy of EPA.

A definitive explanation for the ability of EPA to protect differentiating myotubes from the inhibitory effects of TNF-α is currently lacking. The most widely accepted proposition for its anti-inflammatory efficacy is that EPA changes the membrane phospholipid pool, becoming readily incorporated into the cell membrane at the expense of AA [22,36]. A possible link between our observations and with the activities of EPA reported above can be made by considering that AA is released from cells undergoing TNF-α-mediated apoptosis and that cytoplasmic (c) phospholipase A_2 _(PLA_2_) plays a major role in this process [37]. TNF-α mediates the activation of cPLA_2 _via the TNF-R1. Interestingly, TNF-R1 expression is also upregulated by AA and can be inhibited by ώ-3 PUFAs including EPA [32]. Signalling via the TNF-R1 death receptor is very complex. Simplistically, recruitment of death domain-containing intracellular adaptor molecules at the receptor site transduces apoptotic or survival signals. Thus, recruitment of TNF-R associated death domain (TRADD) and Fas associated death domain (FADD) molecules leads to interactions with, and subsequent activation of, pro-caspase molecules and apoptosis. Alternatively, recruitment of TRAF1 and TRAF2 and their interaction with TRADD leads to activation of Jun N-terminal kinase (JNK) and NF-κB which prevents caspase-8 activation and apoptosis [38]. Since our data showed EPA inhibits caspase-8 activity it would be interesting to investigate this further. Our data also showed reductions in indices of myogenesis in response to TNF-α treatment, including myotube size, myoblast fusion index and expression of MyHC. It has been reported that inhibition of muscle specific protein expression during C2C12 myogenic differentiation by TNF-α is dependent on activation of NF-κB which is a major regulator of inflammatory genes [16,6]. PUFA regulation of gene transcription factors is an emerging area of study and differential effects have been reported; whereas ώ-3 PUFAs inhibit, ώ-6 PUFAs stimulate, NF-κB activation [39]. Similarly, in differentiated human myotubes it was found that saturated fatty acids (palmitate), but not PUFAs, activate NF-κB [40]. The ability of EPA to inhibit NF-κB activation is documented by several studies and seems to involve decreased phosphorylation and thus reduced degradation of the inhibitory IκB complex [22,35]. It has also been suggested that the highly polyunsaturated EPA could be readily oxidised and that oxidised EPA then interferes with NF-κB activation [41]. Peroxisome proliferator-activated receptor (PPAR)γ is a major transcriptional regulator of lipid metabolism whose activity can be inhibited by TNF-α, either by altered PPARγ gene expression or by reduced DNA binding activity. Both types of PPARγ inhibition involve NF-κB activation [42]. Recently it has been shown that altered expression of PPARγ in differentiating C2C12 myotubes inhibited myogenesis [43]. Interestingly, EPA but not other ώ-6 or ώ-3 PUFAs induces PPARγ gene expression in human adipocytes [44].

## Conclusion

In summary, the present study demonstrates that the ώ-3 PUFA EPA exerts a protective effect on differentiating C2C12 myotubes, attenuating the inhibitory effects of TNF-α on indices of myogenesis and apoptosis. These findings are consistent with reports in the literature regarding potential mechanisms of action for EPA, but these now require detailed investigation to clarify the nature of the observed benefits on skeletal muscle cells. These observations suggest for the first time that EPA may have potential benefits in skeletal muscle regeneration.

## Methods

### Cell Culture

The murine skeletal muscle cell line C2C12 was obtained from the European Centre for Animal Cell Culture (ECACC, Porton Down, UK). C2C12 is a well established murine model for studying skeletal muscle differentiation. C2C12 myoblasts are able to undergo differentiation into spontaneously contracting myotubes on withdrawal of growth factors [45]. Thus, myoblasts were cultured in low-glucose Dulbecco's Modified Eagle's medium (DMEM) supplemented with 10% (v/v) fetal bovine serum (FBS) (both from Lonza Biologics, Slough, UK) containing antibiotics (10,000 units/ml penicillin G, 10 mg/ml streptomycin sulfate and 25 μg/ml amphotericin B from Sigma-Aldrich, Poole, UK), referred to as growth media (GM). Myoblasts were seeded at approx.10^4 ^cells/cm^2 ^onto uncoated tissue culture plastic flasks or multi-well plates (Greiner Bio-One, Stonehouse, UK) for 24 h at 37°C and 5%CO_2_, at which point they had reached approx. 70% confluency. At this time myoblasts were induced to differentiate by briefly rinsing cells with PBS and replacing GM with DMEM containing antibiotics, supplemented with 2% (v/v) heat-inactivated horse serum, referred to as differentiation media (DM). For proliferation experiments myoblasts were maintained in GM beyond 24 h, up to 5 days. To evaluate their effects on myogenic differentiation, murine recombinant TNF-α (Peprotech Ltd, London, UK) or EPA (IDS Ltd, Boldon, Tyne & Wear, UK) were added to cell cultures directly following induction of differentiation. EPA was first complexed with fatty-acid free bovine serum albumin (BSA) (Sigma) as described previously [44]. Briefly, EPA stock solutions (50 mM) were prepared in absolute ethanol and stored at -20°C in a glass vial in the dark. Working solutions were prepared by adding the required volume of EPA stock solution to pre-warmed (37°C) DMEM containing 4% (w/v) fatty acid-free BSA. Dilutions were maintained at 37°C for at least 1 h before their addition to cell cultures. The final concentration of ethanol in cultures was always below 0.1%. Myoblasts were cultured in DM for up to 5 days after the treatment(s), receiving fresh media after 48 h, prior to treatment(s). The dosing regimen for EPA and TNF-α experiments was as follows: EPA and TNF-α were delivered immediately following the change to DM and were refreshed with any media changes; EPA treatment was given either as a pre-incubation for 2 h with EPA alone, prior to its removal and replacement with TNF-α alone (EPA pre-treatment) or, as a co-incubation given with TNF-α for the duration of the experiment (EPA co-treatment).

### Assessment of Myogenic Differentiation

To study the effect of TNF-α and EPA treatments on differentiation of myotubes, immunocytochemistry was performed. Cultures were briefly rinsed with PBS, then fixed with ice-cold methanol for 2 min. Cells were then washed three times with PBS for 3 min, blocked with 1% BSA in PBS for 30 min, then incubated with 38 μg/ml of MF-20 antibody (Developmental Studies Hybridoma Bank, University of Iowa, USA) to detect myosin heavy chain (MyHC) protein, at a dilution of 1:250 in 0.5% BSA/PBS for 1 h. After washing with PBS, cells were incubated with 2 μg/ml goat anti-mouse Alexa Fluor^®^-546 IgG, at a dilution of 1:2000 in PBS for 1 h in the dark. After washing with PBS, cells were counterstained with 4',6-diamidino-2-phenylindole (DAPI). The stained cells were analysed under a Nikon TE2000 inverted fluorescence microscope and DAPI and MyHC images were captured with a Hamamatsu Orca camera and merged using Image-Pro Lab v3.7 image analysis software (Nikon UK Ltd, Kingston upon Thames, UK).

Myotube metrics were also quantified using Image-Pro software to determine myotube diameter and a myoblast fusion index. Average myotube diameter was evaluated as the mean of five approximately equi-distant measurements taken along the length of the myotube. For each treatment, 10 fields of view were chosen randomly and 10 myotubes were measured in each field. A myogenic index (MI) was also calculated to indicate myotube fusion and the mean number of nuclei per myotube was calculated to indicate heterogeneity of myotube size. Using 10 images from randomly chosen microscope fields of DAPI and MyHC stained cells for each treatment, the total number of nuclei and the number of nuclei incorporated into myotubes were counted. The MI was calculated as the percentage of nuclei incorporated into myotubes (defined as containing at least two nuclei) relative to the total number of nuclei. Using the same images, the myotube heterogeneity was calculated as the number of nuclei per myotube, within categories of small (2–5 nuclei), medium (6–9 nuclei) and large (>10 nuclei) myotubes, expressed as a proportion (%) of the total myotubes.

### Apoptosis and Necrosis

To determine the contribution of apoptosis to cell death during myotube differentiation, and the effects of EPA treatment on this, cells in DM were stained with Hoechst 33342 to identify apoptotic nuclei and images captured, as described above. Briefly, a final concentration of 1 μM Hoechst 33342 stain (Invitrogen, Paisley, UK) was added directly to cells in culture. After 2–10 minutes media was removed and cells were rinsed in 1× apoptosis wash buffer (Invitrogen) and mounted using one drop of wash buffer and a coverslip. Analysis was performed immediately under fluorescence microscopy. To quantify the extent of apoptosis, an apoptosis index (AI) was calculated as described in [46], from 6 images of randomly selected microscope fields for each treatment. The AI was calculated as the percentage of apoptotic nuclei (brightly fluorescent, condensed compared to normal) relative to the total number of nuclei. As apoptosis during differentiation of myoblasts is characterised by enhanced caspase-8 activation, the effects of TNF-α and EPA treatments on the activity of this apoptosis marker were investigated. Caspase-8 activity from cells in DM was analysed by using a Caspase-Glo 8 assay (Promega, Southampton, UK) performed according to the manufacturer's instructions. Caspase-8 luminescence was quantified on a Tecan Genios reader with Magellan software (Tecan, Reading, UK). A cytotoxicity assay was performed following EPA treatment to determine cumulative cell death over 5 days of culture in DM. Quantitative analysis of cytotoxicity was made using a Cell Titer Blue Assay kit (Promega) and undertaken according to the manufacturer's instructions. Morphological analysis of cellular necrosis was assessed by 0.4% (w/v) Trypan Blue (Sigma) vital dye staining. Myoblasts, seeded at a cell density of 10,000 cells per well in 6-well multiwell plates, were cultured and induced to differentiate as described above. Cellular necrosis was quantified by performing a cell count 48 hours after treatment. The adherent cells were removed by gentle enzymatic detachment with pre-warmed (37°C) Trypsin (Lonza) and combined with the non-adherent cell fraction. Percent viability was determined under phase-contrast microscopy.

### Statistical Analysis

Data is expressed as the mean ± SEM from three independent experiments. Statistical significance between two groups/treatments was tested using unpaired Student's t test. Statistical significance between more than two groups was tested using one-way ANOVA. Statistical significance was set at *p *< 0.05.

## Authors' contributions

PM carried out the experimental studies. SP and JA conceived of the study, and participated in its design and coordination and helped to draft the manuscript. SP performed the statistical analysis. All authors read and approved the final manuscript.
